# Which New Approaches to Tackling Neglected Tropical Diseases Show Promise?

**DOI:** 10.1371/journal.pmed.1000255

**Published:** 2010-05-18

**Authors:** Jerry M. Spiegel, Shafik Dharamsi, Kishor M. Wasan, Annalee Yassi, Burton Singer, Peter J. Hotez, Christy Hanson, Donald A. P. Bundy

**Affiliations:** 1 School of Population and Public Health, Faculty of Medicine, University of British Columbia, Vancouver, B.C., Canada; 2 Centre for International Health, College of Health Disciplines, University of British Columbia, Vancouver, B.C., Canada; 3 Liu Institute for Global Issues, College for Interdisciplinary Studies, University of British Columbia, Vancouver, B.C., Canada; 4 Department of Family Practice, Faculty of Medicine, University of British Columbia, Vancouver, B.C., Canada; 5 Division of Pharmaceutics and Biopharmaceutics, Faculty of Pharmaceutical Sciences, University of British Columbia, Vancouver, B.C., Canada; 6 Global Health Research Program, College for Interdisciplinary Studies, University of British Columbia, Vancouver, B.C., Canada; 7 Emerging Pathogens Institute, University of Florida, Gainesville, Florida, United States of America; 8 George Washington University, Department of Microbiology, Immunology, and Tropical Medicine, and Sabin Vaccine Institute, Washington, D.C., United States of America; 9 United States Agency for International Development (USAID), Washington, D.C., United States of America; 10 The World Bank, Washington, D.C., United States of America

## Abstract

This *PLoS Medicine* Debate examines the different approaches that can be taken to tackle neglected tropical diseases (NTDs). Some commentators, like Jerry Spiegel and colleagues from the University of British Columbia, feel there has been too much focus on the biomedical mechanisms and drug development for NTDs, at the expense of attention to the social determinants of disease. Burton Singer argues that this represents another example of the inappropriate “overmedicalization” of contemporary tropical disease control. Peter Hotez and colleagues, in contrast, argue that the best return on investment will continue to be mass drug administration for NTDs.

## Viewpoint by Jerry Spiegel, Shafik Dharamsi, Kishor Wasan, and Annalee Yassi: A Proportion of Research Funding Should Be Diverted to Addressing Social Determinants of NTDs

The past decade has seen a host of new initiatives and funding to address NTDs affecting the world's poorest people [1]-[4]. But despite this additional funding, the global disease burden remains high [5]. We believe that one of the reasons for this continuing burden is that NTD research has neglected the broad social determinants of disease. We propose a mechanism, a “social offset in research,” to address this neglect.

NTD initiatives have primarily aimed to stimulate drug development by offering incentives for pharmaceutical companies to produce essential medicines for vulnerable populations [6]-[9]. These initiatives have largely ignored other manifestations of neglect, such as the weak health systems and poor socio-environmental conditions that cause and/or perpetuate NTDs. One problem with excessive focus on developing new drugs is that it diverts attention and funding away from complementary strategies needed to sustainably reduce disease burden [10],[11]. Examples of such strategies include (1) improving access to clean water, good sanitation, and adequate housing and (2) community education and mobilization to apply measures needed for effective prevention, such as community-based vector control and training of health workers in infection control measures.

One way to redirect attention and funding toward such complementary strategies is through a type of “social offset” in NTD research. We propose that whenever a research program on an NTD innovation is funded, a proportion of the funding is set aside (“offset”) to address related socio-environmental and health system aspects. The concept of “social offsets” emerged recently in response to the need for affordable housing in some urban areas undergoing gentrification [12]. When low-income residents became unable to continue living in these neighborhoods, developers were asked to pay a “social offset,” i.e. contribute to a fund that invests in affordable housing for low-income people. The idea derives from the notion of “carbon offsets” (to compensate for carbon-producing activities such as air travel, one pays into a fund that mitigates carbon production by investing in clean energy technology or planting trees). In our proposal, we are suggesting that any investment in a narrow biomedical solution is offset by channeling a proportion of that investment into broader approaches for reducing the NTD burden.

We feel this approach is timely, given recent debates on whether vertical (disease-specific) funding has been counterproductive by drawing resources away from public health systems strengthening and preventive measures [13]-[15]. The Commission on Social Determinants of Health [16] has highlighted the importance of “non-medical determinants of health,” but these determinants have yet to be integrated into global strategies for NTD control. Such control requires *both* a biomedical approach and a broader socio-environmental and health systems approach. Our social offsets proposal could be a bridge between the two.

Such integrated approaches are of proven effectiveness, but they have been sidelined recently by over-reliance on biomedical “solutions.” Singer and de Castro note that a century ago the Rockefeller Foundation funded two approaches to hookworm control: treating those infected and addressing the social and environmental determinants of disease by installing sanitary facilities [17],[18]. A similar, integrated approach is now needed for many other NTDs. For example, nobody questions the need for better drugs for visceral leishmaniasis (the focus of coauthor KMW's research) [19], but complementary prevention initiatives, health system strengthening, and improved diagnostics are all equally important for disease control [20]. Yet most research funding in the US for leishmaniasis is devoted to basic research, biomedical innovations, and product development (66.3%), compared to 3.7% for epidemiological research and only 7.1% for implementation research [21].

Merely strengthening incentives to make new medicines more accessible for NTDs [22] still ignores the need for complex health intervention trials that take social and environmental conditions into account and militates against an integrated approach. Recognizing this, the NTD research initiative of our university (University of British Columbia) is explicitly shifting its focus from “drug development and delivery” toward effective disease-reducing interventions [23].

What would our “social offsets” proposal involve? Inspired by the “15 by 2015” campaign that urges donor organizations to allocate 15% of their vertical funding toward sustainable comprehensive primary health care [24], we call for a proportion of NTD research funding to be allocated to financing complex health intervention trials. Specific percentages can vary according to the particular disease and context.

This social offsets approach parallels the way in which some economic development projects have started to consider issues such as sustainability and community well-being. Such projects don't just assess financial costs and benefits, but also consider direct and indirect social and environmental impacts [25],[26]. We believe that it is time to extend this kind of thinking to drug development. Before products are licensed, proponents should not only affirm clinical safety and effectiveness, but also consider the social, environmental, and health systems contexts into which the new drug will be introduced. They should accordingly be required to invest in research that will ensure that introducing their product on the market will have an overall benefit in reducing the burden of the disease in question.

Our proposal builds upon the creative funding initiatives begun by the NTDs movement. It will introduce a mechanism analogous to carbon offsets or to the environmental impact assessments conducted as part of some economic development projects. While much discussion is needed to work out the details, a “social offsets” approach could help conquer NTDs and improve health equity.

## Viewpoint by Burton Singer: Bring Back Primary Prevention for Relieving the Burden of NTDs

The recent designation of a set of tropical diseases as “neglected” [27] has given rise to a groundswell of interest in strategies for their control and research on new tools for alleviation of their burden [28]-[30]. Regrettably, this initiative has also served to expose yet another example of the over-medicalization of contemporary tropical disease control strategies. A primary example is the emphasis on drug administration alone to alleviate the burden of schistosomiasis [31]-[33] and the soil-transmitted helminths, of which hookworm is the most prominent [34]. The extant programs focus on deworming already infected people while doing nothing to prevent reworming of the same individuals. These programs amount to establishing a chain of dependence on drugs with no terminal horizon in sight. The problem is that at some point funds for drugs and their programmatic support fade out [35], and the reworming process escalates afresh.

Reading a bit of history is instructive on this issue. You don't find a demand for drugs to treat hookworm in the southern United States today, because an integrated program of drugs to treat infected cases and installation of toilets (a tool for prevention) as a route for human feces—initially containing hookworm eggs—put an end to the problem almost a century ago. It is worthwhile reading the 1921 annual report of the Rockefeller Foundation and the autobiographical paper by Charles Stiles [36], who ran the hookworm eradication program in the southern US, to get a clear picture of how sanitary engineering can play a basic role in prevention of the corpus of soil-transmitted helminthic diseases on the current NTD list.

Shifting to schistosomiasis, we again have a major drugs-only effort [31]-[33] that could be dramatically improved by cooperative ventures between combinations of the many engineering groups that are currently providing clean water at the village level in the tropics (http://www.globalwaterchallenge.org/home/, http://www.ewb-international.org, http://thewaterproject.org) and the contemporary health personnel who seem to see schistosomiasis control as something to be dealt with by installing an endless chain of dependence on pharmacological agents.

These examples are a useful vantage point from which to re-emphasize a much broader theme: namely, that nearly half of the measurable population-level health improvements in sub-Saharan Africa in the 1990s were a consequence of positive inputs in water and sanitation, housing, transportation, and communication [37]. The focus is on the disease prevention consequences of infrastructure improvements. An important feature of such interventions is that they act on multiple diseases simultaneously. This is of great importance due to the pervasiveness of coinfection in the tropics [38],[39]. Clean water and effective sanitary facilities can simultaneously prevent schistosomiasis and the entire corpus of soil-transmitted helminths on the NTD list. Thus, steps forward toward sustainably reducing the burden of many NTDs are dependent on building bridges between the infrastructure suppliers, rooted in engineering, and the health sector. Financing of reductions in NTDs by preventive measures can be piggybacked onto national infrastructure development, where large sums are involved and where the health sector is presently not engaged.

A useful example of this kind of cooperative venture derives from the concession agreement between the Nam Theun Power Company (NTPC) and the government of Laos PDR [40]. Here a Health Impact Assessment—associated with dam construction and implementation of the Nam Theun 2 Hydroelectric Project—and a Public Health Action Plan [41], together with NTPC implementation of regular health assessments for resettled communities and provision of improved housing with clean water and sanitary facilities, is dealing not only with NTDs, but with the full gamut of health problems of the local people [42]. An important reason for mentioning this direct linkage of infrastructure development to health issues is that it exemplifies the kind of project that currently goes essentially unnoticed in international health circles. However, this was not always the case. Bridge building between corporate development projects in the tropics and public health were facilitated from 1950 to 1978 by a stimulating series of conferences held at the Harvard School of Public Health [43] under the title “Industry and Tropical Health.” These meetings engaged industry representatives with public health people on problems of mutual concern. Regrettably, the vanishing of this series has left a large void for 30 years or more that, under proper leadership, could readily be filled at the present time. The repair of this broken bridge could provide an important base for the financing and implementation of prevention interventions that could substantially reduce the burden of many NTDs.

## Viewpoint by Peter J. Hotez, Christy Hanson, and Donald A. P. Bundy: The Promise of Integrated NTD Control

More than one billion people, mostly in the developing world, suffer from one or more of the neglected tropical diseases (NTDs) [44],[45]. These diseases disproportionately impact poor and rural populations who lack access to safe water, adequate sanitation, and essential medicines. Ninety percent of the global burden of NTDs is caused by a group of seven highly prevalent diseases: onchocerciasis, lymphatic filariasis (LF), trachoma, schistosomiasis, and the three major soil-transmitted helminth (STH) infections (hookworm, roundworm, and whipworm) [44],[45]. In terms of both health impact and cost-effectiveness, few other interventions can rival mass drug administration for NTDs, and increasingly this approach is being recognized for its beneficial effects on strengthening health systems, improving economic development, and achieving the Millennium Development Goals [45].

Considerable progress has been made in the control of the individual diseases through focused programs [44]. Since the 1970s the Onchocerciasis Control Program (OCP) and its follow-on African Programme for Onchocerciasis Control (APOC) have been successful in controlling blindness in sub-Saharan Africa and actually eliminating the disease in Mali and Senegal [44],[46]. Through free drugs provided by Merck (ivermectin, trade name Mectizan) and annual community-directed drug treatments with ivermectin (CDTI) it is projected that by 2010 OCP and APOC will have protected over 150 million individuals from blindness in over 30 countries, and at an economic rate of return expected to reach 18% [47]. Simultaneously, APOC has amassed an army of almost 400,000 volunteer community drug distributors to extend the reach of local health systems [44]. Similarly through mass drug administration of ivermectin or diethylcarbamazine citrate (DEC) together with albendazole (from GlaxoSmithKline) the Global Programme to Eliminate LF has treated almost 2 billion people over the past 8 years, thereby averting 32 million disability-adjusted life years [48], and at a cost of only $14–$30 per DALY averted [49]. The active transmission of LF has also been interrupted in several countries [50]. Through Pfizer donations of azithromycin the International Trachoma Initiative operates control programs in 15 countries [44],[51].

Despite this progress, individuals and communities commonly remain affected by NTDs, as there is considerable epidemiological overlap of these diseases. In more than 75% of countries in Africa, at least six of the seven diseases coexist in some fashion across communities. Today only 9%–21% of children who could benefit from benzimidazole anthelminthics actually receive such essential STH medicines [53], while fewer than 2% of eligible people receive praziquantel for schistosomiasis [44]. School-based deworming programs show particular promise in this role [53]. One of the great public health challenges in the coming decade will be to accelerate the expansion of mass drug administration for STH infections and schistosomiasis, bringing it up to the level of coverage of LF, onchocerciasis, and trachoma, even as these control programs are being extended to reach all at-risk populations.

How then can we ensure sustainability for control or elimination of all of the seven most common NTDs? The solution may, in part, be found in the efficiencies of integrated preventive chemotherapy conducted while longer-term water, sanitation, and development infrastructure is built. Historically, Ministries of Health in disease-endemic countries have supported the control of NTDs through parallel programs. For example, it is not unusual to find a national schistosomiasis control program managed alongside a national LF control program, each with its own plan, funding stream, drug supply chain systems, monitoring and evaluation, and preventive chemotherapy campaigns. If funding was available for one program, it may have been able to implement preventive chemotherapy while its sister program could not. Research has resulted in sufficient evidence to suggest that coimplementation is safe for communities, feasible to implement, and efficient [44]. WHO has endorsed the coimplementation of mass drug administration, an approach also referred to as the integrated approach to preventive chemotherapy [54].

Ultimately sanitation and clean water are also critical for sustaining the impact of control and elimination strategies that rely on mass drug administration, but we must recognize the enormous expense of such interventions and their poor track record in the absence of parallel economic development [55]. Anthelminthic vaccines are also under development, which will contribute substantially to the sustainable elimination of the NTDs [56]. Indeed for some anthelminthic drugs there is also concern about the emergence of drug resistance. However, for at least the next decade we believe that integrated preventive chemotherapy offers the greatest promise, particularly for Africa where NTD burdens are the highest. By linking mass drug administration efforts for the three major STH infections and schistosomiasis with those for onchocerciasis, LF, and trachoma, it should be possible to simultaneously attain high coverage for all seven NTDs [28],[57],[58]. Bundling mass treatments for these conditions could provide cost savings of up to 47% in what is already a low cost-strategy [59]. The integration of NTD control builds on a number of existing strengths including the donation of essential medicines, the successful track record of the major public private partnerships already committed to mass drug administration, the widespread reach of CDTI even in post-conflict countries, and the strengthening of health systems by empowering both health ministries and volunteer community drug distributors [60]. Countries are currently being supported to adopt and scale-up this integrated approach by USAID and other bilateral and private sector donors.

With only 3 years of implementation supported by USAID so far, efficiencies are already being realized and tremendous scale-up of control for the seven NTDs documented. In Sierra Leone, a strong onchocerciasis program treated 12 districts in 2006 and 2007, and children ages 12–59 months were treated for STH infections in 14 districts through Maternal and Child Health Weeks (MCHW) in 2006 and 2007. The Ministry of Health and Sanitation was able to build on the onchocerciasis and MCHW platforms to begin treatment for schistosomiasis and has increased by 117% the number of districts treated for LF and the number of districts treating school-age children for STH infections. In Niger, government commitment to expanding NTD control through an integrated approach has resulted in the number of persons treated for STH infections increasing by 254%, trachoma by 179%, and schistosomiasis by 29%, between 2006 and 2008.

The relatively modest costs of integrated NTD control [56] effectively mean that a little money goes a long way. In 2010 the commitment of $65 million from the United States Government (USG) for integrated NTD control, together with a commitment from the United Kingdom, means that more than 100 million people who would otherwise not have been treated could receive essential NTD drugs next year. Efforts are now in place to establish regional financing mechanisms for NTDs in Latin America and the Caribbean through a trust fund at the Interamerican Development Bank. In Africa, APOC with WHO, World Bank and other development partners is exploring how to integrate CDTI into national health systems and to support country-led coimplementation efforts to control onchocerciasis and other NTDs. Other mechanisms are also under consideration, with similar discussions for Asia underway. More than a decade ago the question was asked “can we deworm this wormy world?” referring to a landmark 1947 paper on the first global prevalence assessment of helminth infections [61]. Integrated NTD control represents the most cost-effective means to achieve global drug coverage and attain this goal, as well as improve maternal and child health, reduce blindness and disability, and ensure elimination efforts in the foreseeable future.
